# Longitudinal amygdala resting state functional connectivity develops differently in adolescents with internalising disorders compared to healthy peers

**DOI:** 10.1192/j.eurpsy.2024.194

**Published:** 2024-08-27

**Authors:** E. Roelofs, J. M. Bas-Hoogendam, N. van der Wee, R. Vermeiren

**Affiliations:** ^1^LUMC-Curium, Department of Child and Adolescent Psychiatry; ^2^Department of Psychiatry, Leiden University Medical Center; ^3^Leiden Institute for Brain and Cognition; ^4^Developmental and Educational Psychology, Institute of Psychology, Leiden University, Leiden, Netherlands

## Abstract

**Introduction:**

Longitudinal neuroimaging studies focused on adolescents with internalising psychopathology (i.e. with clinical anxiety and/or depression) are scarce, even though anxiety and depression are highly prevalent mental illnesses in adolescence. Often linked to comorbidity with anxiety disorders, a large proportion of depressed adolescents displays more severe symptoms and poorer response to treatment. Previous longitudinal resting-state fMRI (RS-fMRI) studies of intrinsic functional connectivity (iFC) in depressed adolescents point to dysregulation of underlying neural networks such as the corticolimbic network, including among others the amygdala and frontal regions, which are involved in emotion processing and regulation.

**Objectives:**

This naturalistic study investigates longitudinal changes in resting-state iFC in adolescents with internalising disorders, compared with healthy peers.

**Methods:**

23 treatment naïve adolescent patients with clinical depression and comorbid anxiety (INT) and 24 healthy controls (HC) participated in RS-fMRI scans at baseline and after three months. Questionnaires measuring anxiety and depression were completed at both timepoints. Imaging analyses were conducted using independent component analysis (ICA) to extract 7 networks, being the default mode, frontoparietal (bilateral), affective, salience, executive control and dorsal attention network. Additional iFC of amygdala subregions, being laterobasal (LB) and centromedial (CM), was investigated using seed-based analyses. To investigate changes over time between groups, voxelwise analyses were conducted using FSL’s PALM.

**Results:**

No significant results within ICA defined networks were found. iFC between the left LB amygdala and left frontal pole significantly increased over time in patients and decreased in HC. iFC between the right LB amygdala and right pre- and postcentral gyrus also significantly increased over time in patients and decreased in HC, and was significantly associated with reduction in depressive symptoms within patients.

**Conclusions:**

This study provides initial evidence that iFC between the laterobasal amygdala and frontal regions develops differently over time in adolescents with internalising disorders compared to healthy peers and that it is associated with reduction in depressive symptoms.

**Disclosure of Interest:**

None Declared

